# Accelerated Detection of Viral Particles by Combining AC Electric Field Effects and Micro-Raman Spectroscopy

**DOI:** 10.3390/s150101047

**Published:** 2015-01-08

**Authors:** Matthew Robert Tomkins, David Shiqi Liao, Aristides Docoslis

**Affiliations:** Department of Chemical Engineering Queen's University, Kingston, ON K7L 3N6, Canada; E-Mails: matthewrtomkins@gmail.com (M.R.T.); david.liao@chee.queensu.ca (D.S.L.)

**Keywords:** micro-Raman spectroscopy, AC electrokinetics, dielectrophoresis, microelectrodes, optical biosensors, M13 phage

## Abstract

A detection method that combines electric field-assisted virus capture on antibody-decorated surfaces with the “fingerprinting” capabilities of micro-Raman spectroscopy is demonstrated for the case of M13 virus in water. The proof-of-principle surface mapping of model bioparticles (protein coated polystyrene spheres) captured by an AC electric field between planar microelectrodes is presented with a methodology for analyzing the resulting spectra by comparing relative peak intensities. The same principle is applied to dielectrophoretically captured M13 phage particles whose presence is indirectly confirmed with micro-Raman spectroscopy using NeutrAvidin-Cy3 as a labeling molecule. It is concluded that the combination of electrokinetically driven virus sampling and micro-Raman based signal transduction provides a promising approach for time-efficient and *in situ* detection of viruses.

## 1. Introduction

Miniaturized surface-based biosensors are a cost effective and portable means for the sensing of biologically active compounds. Although conventional pathogen detection methods are well established, they are greatly restricted by the assay time due to diffusion limitations or the need for incubation [1]. On the other hand, electric field effects generated by microelectrodes embedded into a biosensor's surface can provide the means of overcoming the bottleneck of time-consuming pathogen detection methods. For example, combinations of microelectrode-generated AC electrokinetic effects, such as electro-thermal flows or electroosmosis, and dielectrophoresis (DEP) have been shown to bring about fast convective transport and concentration amplification of a pathogen at a target detection surface [2–4]. Moreover, depending on the frequency of the applied AC field and the electrical/dielectric properties of the pathogen with respect to the medium, pathogens interacting with a microelectrode array while suspended in a biological sample can be attracted to areas of high electric field gradients (a phenomenon called “positive DEP”), or repelled to areas of low electric field gradients (“negative DEP”) [5]. When coupled with a suitable signal transduction method, a microelectrode array able to cause accelerated and deterministic collection of pathogens on a target surface, can play a crucial role in the function of portable and miniaturize biosensors for pathogen detection or identification [4]. Proof-of-principle demonstrations of how dielectrophoresis-assisted preconcentration strategies can enable the detection of a dilute target analyte are numerous. Some characteristic examples are cited here for viruses [2,6], bacteria [7], DNA [8], and peptides [9].

Raman spectroscopy has the potential to act as a “fingerprinting” technique for the identification of pathogens [7]. The resulting spectrograph can be used to identify a target pathogen based on its unique chemical bonds. Improvements in the sensor's sensitivity can be reportedly achieved with the use of surface enhanced Raman spectroscopy (SERS), which for example, can be achieved through the use of metal nanoparticles [10–12]. SERS was also demonstrated in the presence of a roughened metal surface, without the need for a metal particle suspension [13]. Virus detection has been demonstrated on SERS-active substrates fabricated by various methods, such as nano-identation [14], oblique angle deposition [15], or focused ion beam lithography [16,17]. Although they can potentially provide label-free detection of viruses from solutions under certain conditions, these SERS substrate nanofabrication methods are currently non-trivial; hence, their integration with the relatively simple photolithographic process of microelectrode deposition that provides accelerated target capture from solution may substantially increase the manufacturing complexity of a biosensor chip.

Using properly designed planar microelectrodes, the accelerated virus sampling can be focused down to a very small area on the capture surface. The localized concentration amplification effect thus produced makes micro-Raman spectroscopy a very suitable signal transduction method. As a result, an AC electrokinetically enhanced sampling method coupled to an *in situ* micro-Raman spectroscopy would potentially allow for the one-step concentration and detection/identification of viruses from dilute samples. Additionally, depending on the size of the substrate area that is probed, micro-Raman spectroscopy can provide surface mapping capabilities and render a high resolution scan of the capture area in a short time frame. The work presented in this study demonstrates how a biosensor with surface based microelectrodes can be coupled with a micro-Raman spectroscope in order to explore one avenue that can lead to fast detection of viruses.

## 2. Materials and Methods

Unless otherwise specified, all chemicals were supplied by Sigma-Aldrich (St. Louis, MO, USA).

### 2.1. Microelectrode Fabrication and Functionalization

Microelectrodes were fabricated at the Queen's Microfabrication Facility (QFAB). Polished silicon wafers with a diameter of 4 inches and a thermally grown SiO_2_ layer of 0.5 μm thick were purchased from University Wafer (South Boston, MA, USA). The negative photoresist, ma-N 1405 (Microresist Technologies GmbH, Berlin, Germany), was used to photolithographically transfer the microelectrode pattern onto the substrate. A 5 nm layer of thermally evaporated chrome was used to improve the adhesion of the deposited Au layer (100 nm thickness) to the SiO_2_ substrate.

Antibody-functionalized microelectrodes were created by using a procedure adapted from [18]. Briefly, the microelectrodes were immersed in a 3% by volume solution of (3-mercaptopropyl) trimethoxysilane (MTS) in toluene at 80 °C and allowed to react for two hours in a humidity free atmosphere. The electrodes were then washed with toluene and allowed to react for 2 h with, N-γ-maleimidobutyryloxy succinimide (GMBS) (MD Biosciences, Saint Paul, MN, USA) dissolved in 400 μL of dimethylformamide (DMF) and diluted with ethanol to a final concentration of 5 mM. Finally, the microelectrodes were washed with phosphate buffered saline (PBS) and were allowed to react overnight with a 0.6 mg/mL of antibody in PBS solution, after which the substrate was rinsed with PBS. Microelectrodes for use with polystyrene spheres were functionalized with anti-avidin (IgG fraction, produced in rabbit, obtained from Polysciences, Warrington, PA, USA) in PBS solution. Microelectrodes for use with M13 Phage were functionalized with 0.6 mg/mL of anti-fd bacteriophage, used as provided. (Cedarlane, ON, Canada, product number #1001) The functionalized microelectrodes were kept immersed in PBS buffer until used.

### 2.2. Polystyrene Spheres

Polystyrene particles (NAVDY) with a diameter of 200 nm were used as model particles. Polystyrene spheres with a surface modified with NeutrAvidin^®^ and impregnated with a yellow-green fluorescent dye were obtained from Invitrogen (Carlsbad, CA, USA). Stock solutions were diluted with a KCl solution having a conductivity of 880 mS/m to a concentration of 1.0 × 10^8^ particles/mL.

### 2.3. M13 Phage Preparation

Dried samples of M13 phage particles (ATCC# 15669-B1) were purchased from Cedarlane. The M13 phage was re-suspended with 1 mL of Yeast-Tryptone broth. In order to propagate the M13 phage, *Escherichia coli* EMG31 (EGM31) was prepared on nutrient deficient M9 plates. The EMG31 was then transferred to 26 mL of Yeast-Tryptone broth and allowed to reach the log growth phase. 200 μL of log growth EMG31 was then transferred into 20 mL of yeast tryptone broth with 10 μL of the re-suspended M13 phage. After 1.5 h, the 20 mL was separated into centrifuge safe sample holders each containing 2 mL of the solution. After a further 6 h, the samples were centrifuged and decanted. The resulting samples had an approximate concentration of 1.0 × 10^12^ pfu/mL and were stored at 4 °C until needed.

### 2.4. M13 Phage Identification

In order to visually identify the presence or absence of M13 phage on a silicon surface, the following procedure was used. After the application of the electric field, the microelectrodes were washed in phosphate buffered saline (PBS) for 30 min. The microelectrodes were then rinsed in PBS and allowed to react overnight in a 5 mL solution of PBS with 25 μL of biotinylated anti-fd (Sigma-Aldrich, St. Louis, MO, USA, product number B2661), used as received. The microelectrodes were then rinsed with PBS and washed in PBS for 30 min while being gently shaken. The microelectrodes were then placed in a 5 mL solution of PBS with 25 μL of ExtrAvidin-Cy3, used as received. Finally, the microelectrodes were rinsed with PBS, then washed in PBS for 30 min, rinsed in Millipore^®^ filtered water and then washed in Millipore^®^ filtered water for 30 min. Observations and micro-Raman spectra were obtained after allowing the sample to air dry in a loosely closed container with a low-lint tissue.

### 2.5. Pathogen Sampling

**Figure 1.**
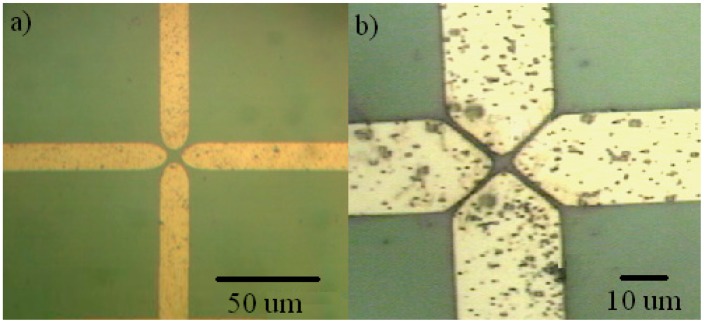
Quadrupolar microelectrodes with (**a**) 10 µm tip to tip spacing between opposite electrodes used for the collection of NAVDY particles and (**b**) 4 µm tip to tip spacing used for the collection of M13 phage.

Non-uniform AC electric fields were generated by sets of planar, quadrupolar gold microelectrodes fabricated on the surface of oxidized silicon substrates, as shown with a top down view in Figure 1a,b. The microelectrodes had a tip to tip electrode spacing of 10 μm for the NAVDY particles (Figure 1a), or 4 μm for the M13 phages (Figure 1b). Power to the microelectrodes was supplied by a signal generator (BK Precision 4040A, Tektronix, Beaverton, OR, USA). The microelectrodes were connected to the source in an alternating fashion (180° phase difference between adjacent electrodes). The value of the applied voltage (V_pp_ = 8 V, peak-to-peak) and applied frequency (f = 1 MHz), were monitored by an oscilloscope (Tektronix 465, Tektronix, Beaverton, OR, USA).

### 2.6. Micro-Raman Spectroscopy

Micro-Raman signals were acquired using a LabRAM (Horiba/Jobin-Yvon, Edison, NJ, USA) with an Olympus BX41 microscope and a HeNe laser source (632 nm). The data were collected and processed using the accompanying software, Labspec v. 4.14-01 (Horiba/Jobin-Yvon). Individual scans were taken using exposure times of 15–120 s, between 15–30 repeats, pinhole sizes of 300 μm, slit widths of 400 μm and a 100× magnification lens.

## 3. Results and Discussion

**Figure 2.**
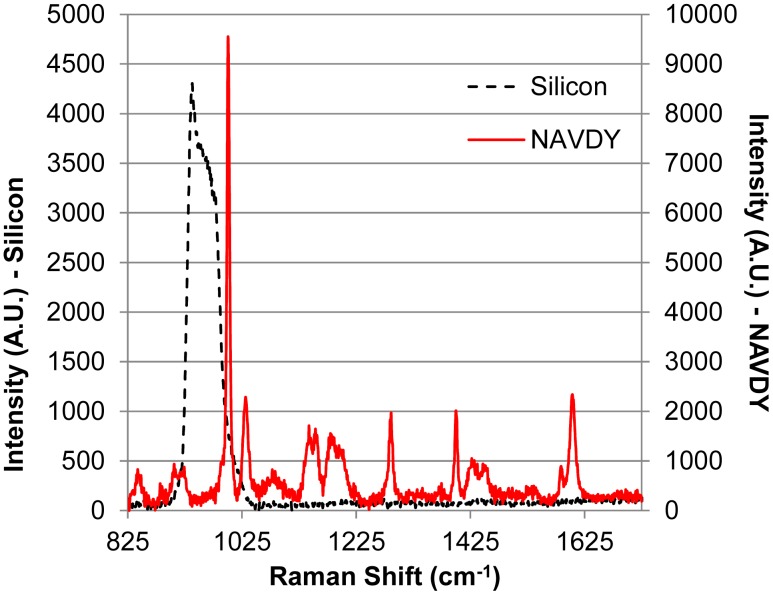
Micro-Raman Spectra of a cleaned silicon surface (black dashed line, left *y*-axis) and a dried, densely packed, photobleached layer NAVDY particles on a glass slide (red solid line, right *y*-axis).

In order to provide some baseline scans for comparison, micro-Raman spectra were recorded for an anti-avidin functionalized silicon surface and from a confluent layer of NAVDY particles dried on a glass slide. The scans for these two substances are presented in Figure 2.

The micro-Raman spectrum for the functionalized silicon substrate for the range between 825 and 1675 cm^−1^ is a relatively flat line with a distinct peak at 938 cm^−1^ and a shoulder in the range of 950 to 1040 cm^−1^. Within the same range analyzed for the functionalized silicon, the NAVDY particles contain several identifiable peaks. A summary of the peaks are presented in Table 1. Seven of the peaks found for the NAVDY particles can be attributed to the in plane vibration modes of the styrene molecule found in the polystyrene polymer of which the particle is composed.

### 3.1. Polystyrene Sphere Mapping

**Figure 3.**
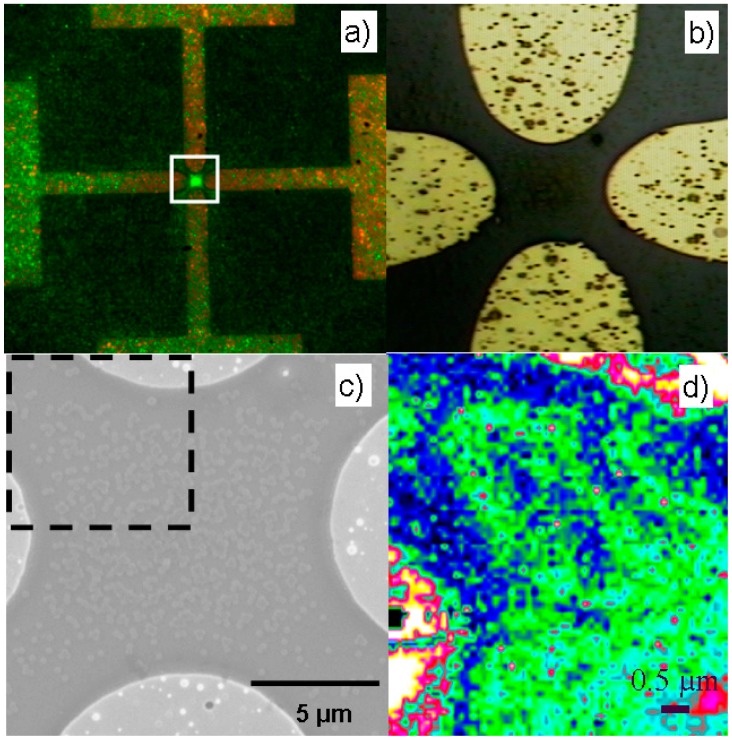
Micro-Raman mapping for the identification of NAVDY particles. (**a**) UV-light image of NAVDY particles captured in the center of microelectrodes; (**b**) Magnified view of the area outlined with a white box in (a) as appears under visible light; (**c**) SEM image of NAVDY particles captured on an anti-avidin functionalized surface; (**d**) Micro-Raman mapping of a portion of the SEM image as indicated by the dashed lines.

Proof of principle studies were first conducted with NAVDY spheres. A typical experimentally observed collection of NAVDY particles is shown in Figure 3 as a bright green dot under UV light. As evidenced by the strong fluorescent signal, the 200 nm spheres collect preferentially at the center of the microelectrode array. The driving force for this collection has been shown to be a combination of negative DEP and viscous forces on the spheres exerted by electrothermal fluid flows [21]. DEP is caused by the direct influence of the spatially non-uniform AC electric field on the polarizable spheres, whereas electrothermal fluid flow stems from the interaction between the electric field and surrounding medium [5].

It is noteworthy that the observed sphere collection begins almost instantaneously. Typically collections, such as the one seen in Figure 3a, occur within approximately 20 s after the AC electric field was turned on. On the other hand, no fluorescent signal was detected anywhere on the microelectrode chip in cases where the electric field remained inactivated, even after long observation times (30 min or longer).

An expanded view of the collection area (white box) is shown in Figure 3b where NAVDY particles can be seen in the centre as they appear under visible light. The white box represents a 20 μm^2^ area that was selected for further investigation. As shown in Figure 3c, the NAVDY spheres collected by AC electrokinetic forces and subsequently captured onto an anti-avidin functionalized surface can be observed in detail with the aid of a scanning electron micrograph. The SEM image depicts a typical particle capture under conditions of negative DEP. Areas of high particle density at the microelectrode centre (area of electric field null) can be seen co-existing with locations devoid of particles, namely the electrode edges and narrow inter-electrode gaps (areas of intense electric field gradients). The micro-Raman mapping results are shown in Figure 3d as the ratio of the intensity of the peak at 999 cm**^−^**^1^ (PS) divided by the intensity of the peak at 1015 cm^−1^ (silicon). The dark blue areas are locations of very low ratio values and effectively indicate the silicon background. The green clusters and the intense red cluster in the bottom right are areas of high ratios and indicate the location of the NAVDY spheres. This ratio has a maximum value at the centre of the electrodes where the sphere concentration is expected to be the highest. The gold electrodes are visible as yellow/white areas at the top right and bottom left. Figure 3d demonstrates how closely the micro-Raman system reproduces the location of the NAVDY spheres on the surface of microelectrode surface and its consistency with the expected negative DEP collection pattern. In the bottom right, the spheres are more closely packed. At the electrode edges, particles are pushed away due to the negative DEP force and a band of silicon is present. Closer to the electrode channel, fewer particles are found and the silicon substrate becomes more prominent. This mapping is an example of how the micro-Raman spectroscopy detection principle could be integrated with AC electrokinetically driven collection in future surface based biosensors for the detection of pathogens.

### 3.2. M13 Phage Detection

M13 phage is a biohazard level 1 pathogenic particle that infects *E. coli* and has commercially available antibodies. M13 phages are filamentous and are generally 7 nm in diameter and 900 nm in length [22]. Literature has reported on the Raman spectra of the fd phage, a phage from the Ff class which share structural similarities with the M13 phage like the size and structure of the DNA loop and the 2800 GP8 coat proteins which make up the majority of its surface [23]. The GP8 protein is the complimentary antigen to the anti-fd antibody used in this research. Previous studies for determining the micro-Raman spectrum of the M13 phage used 10 μL samples with concentrations ranging from 50–80 mg/mL [23]. With an average molecular weight of 1.64 × 10^7^ Da, this corresponds to a concentration of approximately 2 × 10^15^ particles /mL [24].

The applicability of the detection principle established in the previous section for polymeric nanospheres was further explored for the case of M13. However, it was found that successful M13 phage capture under conditions of negative DEP was not possible. This was attributed to the competing effects between DEP and the strong electrothermal fluid flow that arises in suspending media of high electrical conductivity (here, 880 mS/m) [21]. For this reason, M13 collection was attempted by means of positive DEP. To enable positive DEP of M13, the original virus stock suspension was diluted 1000-fold with DI water. The resulting sample conductivity was 1.6 mS/m and the corresponding M13 phage concentration was equal to 1.0 × 10^9^ pfu/mL.

Surface enhanced Raman spectroscopy has been shown to enhance the signal obtained for biological molecules in the presence of nanometer sized metal particles [25] or when on a roughened metal surface [13]. This principle was explored by first collecting M13 phages via positive DEP onto an anti-fd functionalized surface and subsequently allowing a droplet of a 100 nm colloidal gold suspension to dry on the captured virus. It was found that the presence of the colloidal gold enabled some peaks to increase in relative intensity; however, all peaks were matched to a Raman spectrum acquired from an antibody functionalized microelectrode array that did not contain M13 phage.

The inability of this approach to detect the presence of virus first raised the questions of whether the intensity of the electrokinetic effects is sufficient to cause virus collection from the bulk of the solution. Simulations performed on the spatial distribution of the dielectrophoretic force on the virus (not shown here) indicated that the expected dielectrophoretic force acting on the M13 phage in the vicinity of the microelectrodes, modeled as a prolate ellipsoid, would be approximately 7.61 fN. This force is relatively small, considering that the calculated forces acting on vesicular stomatitis virus that was captured successfully in a previous experimental study [2] was on the order of 220 fN. However, results from [3] suggested that the threshold force required to overcome Brownian for a virus of comparable size and aspect ratio to that of the M13 phage, is 3.16 fN. Theoretically, the conditions here should enable the collection of M13 phages.

**Figure 4.**
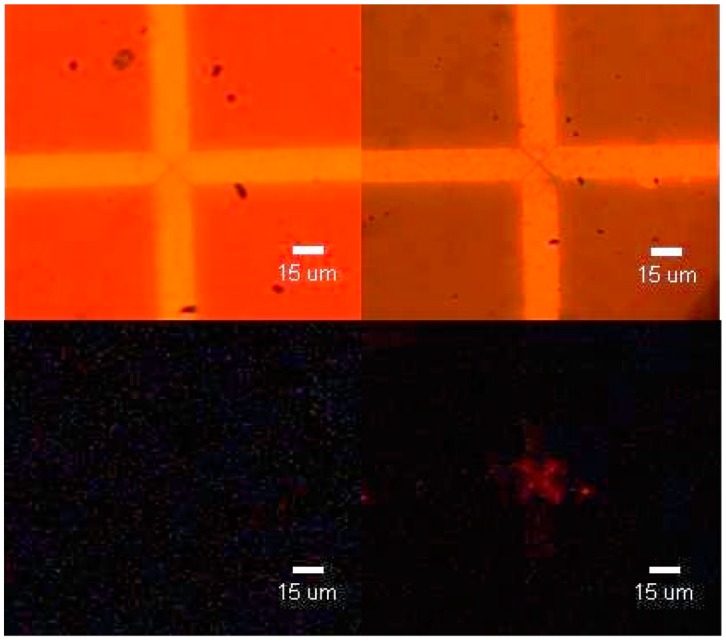
Demonstration of M13 capture using triangular electrodes with a gap spacing of 4 μm. Left: Visible light (**top**) and UV light (**bottom**) images of anti-fd functionalized electrodes. Right: Visible light (**top**) and UV light (**bottom**) images of anti-fd functionalized electrodes with dielectrophoretically captured M13 phage. Conditions: V_pp_ = 15 V, f = 1 MHz, capture time: 30 min.

A tagging method was subsequently used to confirming the presence or absence of M13 phage as outlined in Section 2.4. A comparison is presented in Figure 4 between a control sample without M13 phage (left) and a sample with M13 collected under the previously outlined conditions (right). The control demonstrates that the presence of an electric field does not cause any degradation of the electrodes, nor indicated a false positive. The collection of M13 phage is shown by the red shaped “X” and is consistent with the theoretically expected virus collection pattern undergoing positive dielectrophoresis, *i.e.*, collection near the electrode edges. These results prove that M13 collection at the tips of the microelectrodes can be achieved under the experimental conditions used in the present study.

**Figure 5.**
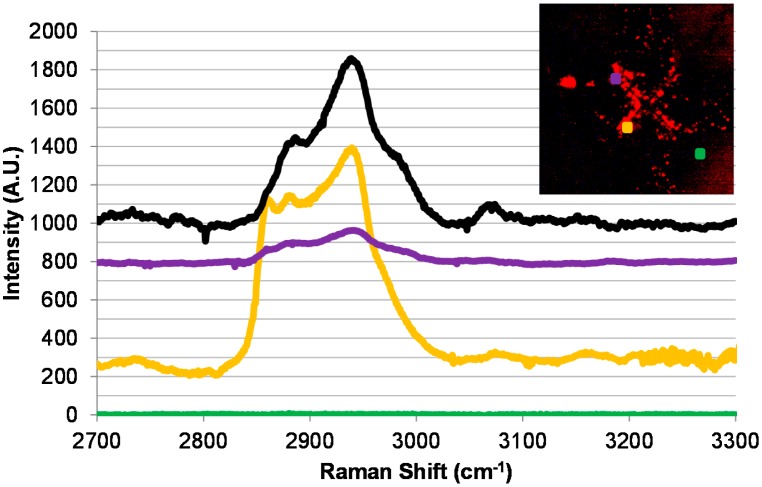
Micro-Raman spectra collected from an anti-fd functionalized microelectrode with captured M13 phages bound to biotinylated-anti-fd reacted with ExtrAvidin-Cy3 prior to photobleaching. The inset in the top right is a magnified image of the collection presented in Figure 4. Black line (positive shifted by 1000 A.U.): a photobleached sample of ExtrAvidin-Cy3 on Silicon. Purple line (positive shifted by 800 A.U.): a scan from the top left point in the inset (indicated by the purple dot). Yellow line (positive shifted by 250 A.U.): a scan from the bottom left point in the inset (indicated by the yellow dot). Green line: a scan from the bottom right point in the inset (indicated by the green dot).

The collected virus was subsequently subjected to a micro-Raman investigation. The inset in Figure 5 indicates three different locations where micro-Raman scans were done: a ‘purple’ location in the upper left of the “X”, a ‘yellow’ location closer to the centre of the “X” in the bottom left, both locations where the presence of M13 is expected, and a ‘green’ location away from the centre of the microelectrodes where no M13 phage is expected to be present. The micro-Raman spectrum of the NeutrAvidin-Cy3 compound is shown in Figure 5 in black and is offset by 1000 A.U. The most distinct peaks occur from 2830 to 3024 cm^−1^ with maxima at 2885 and 2940 cm^−1^ and a shoulder at 2983 and 3010 cm^−1^. A wide peak is also present from 2700 to 2765 cm^−1^ and a narrower one present at 3070 cm^−1^. As expected with positive DEP, the ‘green’ location away from the electrode tips shows no indication of any NeutrAvidin-Cy3 being detected by the micro-Raman scans. The ‘yellow’ and ‘purple’ scans present in the figure are offset by 200 and 800 A.U respectively and differ in their relative intensity. Both sets of scans have peaks at 2940 cm^−1^, 2880 cm^−1^ and at 2860 cm^−1^ which are attributed to methyl stretching. These bands are also present on the NeutrAvidin-Cy3 scan, although the relative intensities may be different due to molecular orientation [26]. Furthermore, the yellow scan shares the same wide peak from 2700 to 2765 cm^−1^ and the narrower one present at 3070 cm^−1^ that are similarly found in the NeutrAvidin-Cy3 scan. The similarities of these scans indicate the successful detection of labeled M13 phage on a micro-Raman coupled AC electrokinetically enhanced surface-based biosensor. Unfortunately, due to long times required for point detection (approximately 8 min), a surface mapping of the inter-electrode area (as in Figure 3d) was not attempted.

The difference in the intensity of the purple and yellow scans in Figure 5 highlights an important consideration when designing microelectrodes for detection with a coupled micro-Raman system. The intensity of a peak is related to the concentration of the analyte in the scanned point. Therefore, if the confirmation is based solely on intensity, information may be lost and the scans misinterpreted. However, the use of relative intensity, as shown in Figure 2 for the NAVDY particles, would allow for the accurate identification of the target particle regardless of absolute intensity of a single peak.

While the M13 phage may be probed via micro-Raman spectroscopy without the need for a labeling agent [23], in the present case the concentration of the phage at the electrode edges was not sufficient to enable such detection. Furthermore, attempts at employing the SERS principle or increasing exposure times did not result in label-free detection of the phage. An obvious improvement of the present detection method, which will also render it “one-step” (labelling-free), would be the incorporation of a SERS active substrate in the area where enhanced virus capture is expected to take place, *i.e.*, in the vicinity of the microelectrode tips. Such integration will likely present challenges both from micro/nano-fabrication and operation point of view since the noble metal of the SERS active layer and the microelectrode layer must be deposited sequentially and remain electrically separated. Our research group is currently investigating experimental procedures that can accomplish this integration. Thankfully, recent technical reports provide a multitude of options on how a SERS active surface can be produced (see, for example, [27]).

In addition to signal amplification via SERS, an efficient dielectrophoretic preconcentration method is also necessary for the successful detection of viruses from dilute samples. Although the small tip-to-tip distance between microelectrodes used in the present study can generate a DEP force strong enough to capture viral particles, the trapping effect of this force barely extends more than a couple of microns away from the microelectrode tips. A more efficient virus capture strategy must take advantage of a favorable convective virus transport to the vicinity of the dielectrophoretic trap. This can be accomplished, for example, by means of electroosmosis in low conductivity samples, carefully implemented electrothermal forces in high conductivity samples, or the introduction of an externally driven microfluidic flow regime over the microelectrode array.

## 4. Conclusions

A demonstration of how AC electric field effects can be paired with micro-Raman spectroscopy toward the successful and time efficient detection of labeled M13 virus particles has been presented. AC particle electrokinetics was shown to accelerate the focusing and capture of sub-micrometer sized bioparticles in antibody-decorated planar microelectrode arrays within only 20 s for the case of NeutrAvidin^®^ particles (200 nm) and 30 min for the case of M13 phage. Micro-Raman spectroscopy was found to be a suitable technique for detecting the captured and Cy3-taged virus. Additionally, the capabilities of micro-Raman spectroscopy to produce spatially resolved scans that reveal the surface distribution of the captured target was shown for the case of NeutrAvidin^®^ particles. The results acquired herein from the coupling of particle electrokinetics with micro-Raman spectroscopy in an experimental set-up akin to a surface based biosensor provided insights for future designs and generated hope that this type of optical biosensors can one permit the timely and *in situ* detection of pathogenic particles that would otherwise remain undetectable.

## Figures and Tables

**Table 1.** Summary of identified peaks for NAVDY particles presented in Figure 2.

**Wavenumber (cm^−1^)**	**Attributed to**	**Reference**
840	Polystyrene	[19]
885	-	-
908	-	-
915	Polystyrene	[19]
999	12 A1—Ring Breathing	[19,20]
1028	18a A1—Ring Breathing	[19,20]
1141	Polystyrene	[19]
1150	15 B2—Ring Breathing	[19,20]
1182	9a A1—Ring Breathing	[19,20]
1200	Polystyrene	[19]
1284	-	-
1397	-	-
1429	-	-
1447	19b B2—Ring Breathing and δCH_2_	[19,20]
1585	8b B2—Ring Breathing	[19,20]
1603	8a A1—Ring Breathing	[19,20]
